# MIP-3β/CCL19 is associated with the intrathecal invasion of mononuclear cells in neuroinflammatory and non-neuroinflammatory CNS diseases in dogs

**DOI:** 10.1186/1746-6148-10-157

**Published:** 2014-07-12

**Authors:** Janina Bartels, Brett G Darrow, Scott J Schatzberg, Lijing Bu, Regina Carlson, Andrea Tipold

**Affiliations:** 1 Department of Small Animal Medicine and Surgery, University of Veterinary Medicine Hannover, Buenteweg 9, Hannover 30559, Germany; 2 Lauderdale Veterinary Specialists, 3217 NW 10th Terrace, Fort Lauderdale, FL, USA; 3 VESC Veterinary Emergency and Specialty Center, 2001 Vivigen Way, Santa Fe, NM 87505, USA; 4 Biology Department, University of New Mexico, 1 University Boulevard, Northeast, Albuquerque, NM 87131, USA

**Keywords:** Chemokine, MIP-3β/CCL19, Steroid responsive meningitis-arteritis, Meningoencephalomyelitis of unknown origin, Intervertebral disc disease, Chemotaxis

## Abstract

**Background:**

Chemokines such as MIP-3β/CCL19 are important factors in the mechanism of cell migration and pathogenesis of central nervous system (CNS) inflammatory reactions. The hypothesis of this study is that CCL19, also known as MIP-3β, is involved in the pathogenesis of inflammatory and non-inflammatory CNS diseases of dogs. Experiments were performed on cerebrospinal fluid (CSF) and serum samples of dogs affected with steroid responsive meningitis-arteritis (SRMA) during the acute phase as well as during treatment. Dogs with SRMA were compared to dogs with presumed meningoencephalomyelitis of unknown origin (MUO), and both groups sub-categorized into dogs receiving no therapy and with patients receiving prednisolone therapy. Idiopathic epilepsy (IE), a group with normal CSF cell count, was used as a control. Additionally, dogs with intervertebral disc disease (IVDD) of varying severity were analyzed. Chemokine concentrations were determined by enzyme linked immunosorbent assay. Migration assays were performed on seven selected CSF samples using a disposable 96-well chemotaxis chamber.

**Results:**

CCL19 was detectable in CSF samples of all dogs. Dogs with untreated SRMA and MUO displayed pronounced CCL19 elevations compared to the control group and patients receiving glucocorticosteroid treatment. CSF cell counts of untreated SRMA and MUO patients were significantly positively correlated with the CCL19 CSF concentration. IVDD patients also had elevated CCL19 concentration compared to controls, but values were considerably lower than in inflammatory CNS diseases. Selected CSF samples displayed chemotactic activity for mononuclear cells in the migration assay.

**Conclusions:**

CCL19 CSF concentrations were markedly elevated in patients affected with the neuroinflammatory diseases SRMA and MUO and showed a strong correlation with the CSF cell count. This chemokine may play an important role in the pathogenesis of SRMA and MUO. The elevation of CSF CCL19 in IVDD suggests that it may also be involved in the secondary wave of spinal cord injuries.

## Background

In several neurologic diseases, a pleocytosis is often detected in the cerebrospinal fluid (CSF). Lymphocytes and plasma cells have been shown to prevail in the cell count in viral infections [1], chronic steroid responsive meningitis-arteritis (SRMA) [2], granulomatous meningoencephalomyelitis (GME) and in necrotizing encephalitides (NE) [1,3]. A neutrophilic pleocytosis is characteristic for bacterial infections and the acute stage of SRMA [2]. A mixed cell population can be seen in protozoal diseases, chronic bacterial infections, necrotic lesions, and in GME [1]. In addition to inflammatory lesions, mild mixed cell pleocytosis can also be seen with acute intervertebral disc disease resulting in central nervous system (CNS) myelomalacia or infarction [4]. Chemokines are important factors in the mechanism of cell migration and thus play a relevant role in the pathogenesis of inflammation within the CNS [5]. They up-regulate surface molecules on circulating inflammatory cells in the periphery which enable them to more efficiently adhere to the endothelial cells of the blood–brain barrier (BBB) and react to the CNS chemokine gradients [6]. One aim of therapeutic research is to develop specific treatments to modulate the inflammatory response within the CNS [7] in diseases such as SRMA and meningoencephalitides of unknown origin (MUO), and also modulate inflammation in intervertebral disc diseases which may be responsible for secondary wave injury [8-10]. One chemokine that could be involved in the pathogenesis of an inflammatory reaction and is a compelling candidate for future modulation is (C-C motif) ligand 19 (CCL19), also known as MIP-3β (Macrophage Inflammatory Protein- 3 beta). This protein will be referred to as CCL19 for most of the text of this manuscript but, these terms may be used interchangeably. CCL19 is constitutively expressed in the CNS for fast immunosurveillance [11] and is produced by different cells such as dendritic cells (DC), macrophages and some non-hematopoietic cells [11,12]. It binds to the CCR7 receptor which is expressed on myeloid cells [13], mature DC, T cells, as well as activated B cells [14-16]. CCL19 was suggested to be involved in the development of chronic inflammation and lymphoid neogenesis, guiding B cells and T cells into the target organ, such as the brain [16]. It is also expressed de novo in various neuroinflammatory diseases such as multiple sclerosis [11] or chronic experimental autoimmune encephalomyelitis (EAE) [16,17]. Bone marrow derived microglia or macrophages connect the brain with the immune system [11,18]. Guiding these cells to the CNS may also be regulated by CCL19, due to its role in attracting macrophage progenitors [11,19]. Microglia may produce CCL19 and were shown to be activated in canine diseases such as MUO [20] and spinal cord injury [10]. In the current study, our hypothesis is that protein CCL19 is, at least in part, involved in the pathogenesis of CNS diseases and may be involved in the invasion of mononuclear cells into the subarachnoid space of dogs. Therefore, three diseases were chosen: steroid-responsive meningitis-arteritis (SRMA), meningoencephalomyelitis of unknown origin (MUO), and intervertebral disc disease (IVDD). In SRMA, a Th2-mediated immune response was shown [21,22] and a Th17 skewed immune response may be accountable for maintaining the inflammatory reaction [23,24]. For comparison, MUO was chosen as a disease group with a predominantly mononuclear pleocytosis within the CSF [1,25,26] despite the heterogeneity of the group. Examples of idiopathic inflammatory disorders of the CNS are NE and GME, in which a strong genetic component [27], occurrence of autoantibodies [28], and the distribution of lymphocytes and macrophages [29], suggest a strong involvement of mononuclear cells in the pathogenesis. A third group was analyzed. In spinal cord injury involving intervertebral disc herniation, a local inflammatory reaction has been documented [30] and the macrophage count within the CSF is thought to be positively associated with the degree of spinal cord damage [31]. With this investigation, we seek to understand the role of CCL19 in the aforementioned CNS diseases in dogs and establish whether this chemokine is generally associated with CNS inflammation or specific to certain diseases.

## Methods

### Samples from patients and controls

A total of 141 patients were analyzed following referral to the Department of Small Animal Medicine and Surgery, University of Veterinary Medicine Hannover, Germany and the Veterinary Emergency and Specialty Center (VESC) Santa Fe, NM, USA . The study was approved by the ethics committee of the University for Veterinary Medicine Hannover. The experimental protocols and procedures in healthy dogs were performed in accordance with the European Communities Council Directive of 24 November 1986 (86/609/EEC) and were approved by the authorities in Lower Saxony (animal experiment number 33.42502/05-12.05). CSF and serum samples for CCL19 assays were obtained and stored at -20°C. Blood was collected via cephalic, saphenous or jugular venipuncture into serum tubes. CSF collection was performed under general anesthesia by cisternal puncture for SRMA, MUO, IE and healthy dogs or lumbar puncture for IVDD dogs. The CSF was centrifuged and the sediment was removed. The remaining CSF was stored at -20°C within 2 hours of sample collection. Number of patients and their laboratory findings are provided in Table 1.

**Table 1:** CSF and serum characteristics in patients with IE, neuroinflammatory disease, IVDD and clinically healthy patients

	**SRMA untreated**	**SRMA treated**	**MUO untreated**	**MUO treated**	**IVDD 2/3**	**IVDD 4/5**	**Healthy**	IE (control)^*^
**No. patients**	25(5/4/16)	26	16	11	25	11	6	21
**CSF cellcount/3microL**	528 (200–3800)^a^	1 (0.25-2)	196 (25.8-73 1.8)^b^	2 (1–3)	1(1–3)	3 (2.5-4)		1 (0–1)
**CSF protein mg/dl**	28 (20–55)^c^	12 (11–14)	40 (25.3-97.3)	16 (14–20)	19(15–22)	14(10.5-16)		12 (10–14)
**CSF Ig A**	1.6 (0.4-2.7)	0.2 (0.09-0.45)						
**Serum Ig A**	184 (106–282)	75.4 (47.9-98.7)						
**Blood leucocvtes**	20.7 (18–27)	10.9(9.8-13.9)	10.6(9.7-13.1)	10 (9.7-12)	8(7.5-11)	13.2(8.3-15.7)	10(9–12)	10 (9–12)
xl000/microL								
**CSF CCL19 pg/ml**	1690 (671.5-3200)^* 'ac^	80.5(68–111.5)^'^	373.5 (174.5-776.8)^* ^ b^	91.8 (76.695)^^^	108.2 (82–162)^* d^	149.2 (97.6-207)^*^	51.2 (40.1-54.2)^*^	66(56–87.1)
**Serum CCL19 pg/ml**	129.3 (73–198)	84.5(35.5-131.9)	160.6 (79.7-467.9)	51.8 (32.4-190)	146.4 (73–191)^d^	97.6 (47–129.3)	108.9 (90.4-192)	88.3 (55.8-127)

SRMA = steroid-responsive meningitis-arteritis; MUO = meningoencephalomyelitis of unknown origin; IVDD = intervertebral disc disease; IE = idiopathic epilepsy; Ig = immunoglobulin; No = number; CSF = cerebrospinal fluid; pg = picogram; microL = microliter; ml = milliliter; mg = milligram; SRMA untreated: n = 25 total; n = 5 one time pre-treated; n = 4 relapse; n = 16 untreated.

^*^Statistically significant differences, CSF CCL19 comparison SRMA, MUO, IVDD, healthy with IE (control).

^'^Statistically significant differences, CSF CCL19 comparison SRMA untreated with SRMA treated.

^^^Statistically significant differences, CSF CCL19 comparison MUO untreated with MUO treated.

^a^Statistically significant differences, correlation SRMA CSF cell count with CSF CCL19.

^b^Statistically significant differences, correlation MUO CSF cell count with CSF CCL19.

^c^Statistically significant differences, correlation SRMA CSF protein with CSF CCL19.

^d^Statistically significant differences, correlation IVDD CSF CCL19 with Serum CCL19.

Data are provided as medians (25th- 75th percentiles).

SRMA group: Fifty one patients were diagnosed with SRMA based on clinical signs, complete blood and CSF examinations, normal cervical radiographs, elevated IgA levels in CSF and serum, response to glucocorticosteroid treatment. The untreated SRMA group consisted of 25 patients which received no medication at the time of sample collection. Nine of these dogs were evaluated separately, five patients having received a one-time pre-treatment with corticosteroids by the referring veterinarian and four patients having relapsed after stopping previous therapy. CSF and blood findings are summarized in Table 1. The SRMA treatment group consisted of 26 of the 51 patients having received more than one steroid treatment prior to CSF acquisition.

MUO group: CSF was also obtained from 27 patients with presumed MUO. Sixteen of these patients received no therapy (untreated group), while 11 patients were already being treated with prednisolone (treated group) (Table 1). The suspected diagnosis was supported by clinical signs, CSF analysis (predominantly mononuclear pleocytosis) and magnetic resonance imaging (MRI) findings employing T1W, with and without contrast medium, T2W, and fluid attenuated inversion recovery (FLAIR) sequences. In ten patients, the diagnosis was confirmed by histopathological examination and included four cases of GME, two of NE and four cases with MUO with a histopathological pattern not related to GME or NE.

IVDD group: The CSF of 36 patients with IVDD was analyzed. The diagnosis of IVDD was made by clinical signs, CSF analysis, MRI and surgery findings. Based on the severity of clinical signs, the patients were grouped into five grades [32]. Twenty five patients were combined with the neurologic status grade 2/3 to one group. The dogs were mild to severely paretic, but capable of spontaneous movement. A second group contained 11 patients with the neurologic status grade 4/5, which were paralyzed with or without deep pain sensation (Table 1).

Control group: CSF samples with normal reference values (less than 5 white blood cells/μl, cisternal protein less than 25 mg/dl) [1] were analyzed. Twenty one patients were diagnosed with idiopathic epilepsy based on clinical signs, blood work, MRI and CSF results (Table 1). IE, a non-inflammatory CNS disease with normal CSF cell count, was compared to SRMA, MUO and IVDD.

Healthy dogs: Additionally, six samples were obtained from healthy dogs (Table 1) (Animal Experiment number: 33.42502/05-12.05) and compared to dogs affected with IE to evaluate differences of CSF CCL19 concentrations between dogs with normal CSF cell counts.

### Measurement of CCL19

Concentrations of CCL19 in the CSF and serum were determined by sandwich ELISA (Enzyme linked immunosorbent assay). An ELISA Kit specific for canine CCL19 (E90096Ca 96 Tests) was used according to manufacturer’s instructions (Uscn Life Science Inc., Wuhan, P.R. China). Briefly, the microtiter plate provided in the ELISA kit has been pre-coated with a monoclonal antibody specific to CCL19. CSF samples were used undiluted or were diluted to a dilution of 1:13 with the standard diluent depending on the CCL19 output concentration. Serum samples were used undiluted for the ELISA. Seven wells were prepared for the dilutions of standard, one well was left blank and the rest of the 96 wells were used for the CSF and serum samples. Four CSF and serum samples of the IE group served as a control and one of these IE samples was pipetted to the plates in each experiment. Samples as a positive control were CSF and serum samples of the neuroinflammatory group and the same sample was used in each experiment at same dilution. The color change was measured spectrophotometrically at a wavelength of 450 nm ± 10 nm on a microplate reader equipped with the analysis software Gen 5 (Synergy2 HT multi-mode microplate reader, BioTek Instruments Inc., Bad Friedrichshall Germany). The detection range of this method was 15.6 - 1000 pg/ml. The standard curve was created using a lyophilized standard reagent (Uscn Life Science Inc., Wuhan, P.R. China) provided in the ELISA kit. Standard concentrations used for the ELISA were 1000 pg/ml, 500 pg/ml, 250 pg/ml, 125 pg/ml, 62.5 pg/ml, 31.2 pg/ml, 15.6 pg/ml. The minimum detectable dose of canine CCL19 is 6.5 pg/ml. All measurements of the CSF and serum were performed in duplicates.

### Chemotaxis assay

To evaluate chemotactic activity of measured CSF samples for mononuclear cell migration, assays were performed using a disposable 96-well chemotaxis chamber with polycarbonate filters (5 μm pore size, 3.2 mm diameter size, 30 μl well size) (Chemo TX Disposable Chemotaxis system, NeuroProbe, Gaithersburg, MD). CSF samples of seven patients were examined, three untreated patients diagnosed with SRMA, two untreated patients with the suspected diagnosis of MUO and two patients with IVDD. Peripheral mononuclear cells (PMCs) were separated from five milliliters of fresh EDTA blood collected via cephalic venipuncture of a healthy dog. PMCs were separated by Ficoll gradient centrifugation according to the technique described by Toth et al. 1992 [33]. PMCs were washed twice with ten milliliters Hank’s balanced salt solution (HBSS) (Fa. Sigma-Aldrich, Deisenhofen, Germany) and re-suspended in Cell-Wash fluid (Becton Dickinson, Heidelberg, Germany). The cell suspension was stained by trypanblue (0.4% in phosphate buffered saline (PBS) Fa. Sigma-Aldrich, Schnelldorf, Germany) and counted in a TURK cell counting chamber (Fa. Brand, Wertheim, Germany). Twenty five microliters of the cell suspension (50000 cells/25 μl) were pipetted directly on the top side of the filter, which is coated with a hydrophobic mask around each of the test sites. The hydrophobic mask restricts the cell suspension to these sites, eliminating the need for upper wells. A known serial dilution of the cell suspension with 50000 cells, 25000 cells, 12500 cells, 6250 cells, 3125 cells, 1563 cells and 781 cells diluted in medium was pipetted in one row of wells to serve as a standard with which to compare the readings from the experimental wells. The rest of the 96 wells were loaded in duplicates with 29 μl of SRMA (untreated n = 3), IVDD (n = 2) and MUO (untreated n = 2) CSF samples diluted in equal volume in Rosewell Park Memorial Institute (RPMI)1640 medium with L-glutamine (Gibco^®^ RPMI Media 1640) containing 1% bovine serum albumin (BSA) and a negative control of pure medium. The loaded chamber with added cell suspension on the top side of the filter was cultured at 37°C in humidified air with 5% CO2 in an incubator (SANYO Sales & Marketing Europe GmbH, Munich) while the migration time for the cell suspension was measured at 30 minutes, 1 hour, 1.5 hours and 2 hours. The filter was removed and cells that had migrated through the filter to the lower wells were counted by alamarBlue^®^ assay (Serotec, Düsseldorf, Germany). AlamarBlue^®^ reagent was added as 10% of the sample volume, followed by 1 to 3 hours incubation at 37°C. Resazurin, the active ingredient of alamarBlue^®^ reagent, is a non-toxic, cell permeable compound that is blue in color and virtually non-fluorescent. Upon entering cells, resazurin is reduced by viable cells to resorufin, a compound that is red in color and highly fluorescent. The resulting fluorescence was read on a microplate reader equipped with the analysis software Gen 5 (Synergy2 HT multi-mode microplate reader, BioTek Instruments Inc., Bad Friedrichshall Germany).

### Statistics

For group comparison, the Kruskal-Wallis test and the Mann–Whitney–Wilcoxon tests were used. Using the Shapiro-Wilk test, an abnormal data distribution was found. Correlation between two variables were tested by Spearman and Kendall rank correlation tests. Statistical significance was set at the 5% level (P < 0.05).

## Results

### CCL19 concentration in CSF of the control group (idiopathic epilepsy) and healthy dogs

**Figure 1:**
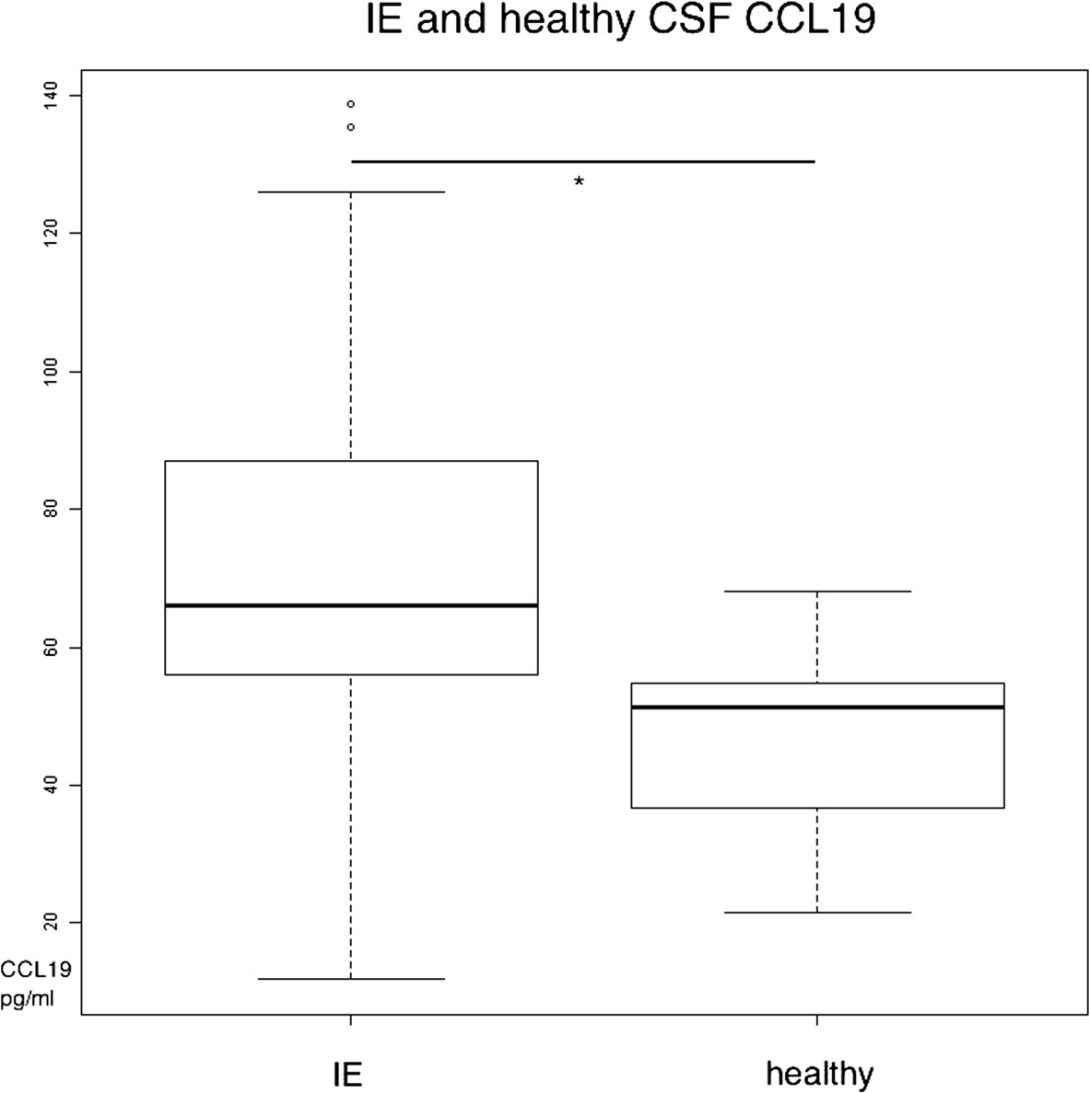
**CCL19 CSF concentrations of patients with IE and healthy animals.** Boxes contain values from the 1st to the 3rd quartile, lines inside boxes indicate median values, endpoints of vertical lines display minimum and maximum values, o represents the outliers. Asterisks indicate statistically significant differences (*P < 0.05; **P < 0.01; ***P < 0.005). IE = idiopathic epilepsy; CSF = cerebrospinal fluid; pg = picogram; ml = milliliter.

CCL19 was measurable in all 21 samples of patients with idiopathic epilepsy and in the 6 samples from healthy donors (Table 1). The comparison between IE and healthy animals showed significant difference (p = 0.0488) which is shown in Figure 1. Although there were significant differences detected between samples from dogs with IE and from healthy animals, IE is considered as the control group due to normal CSF examinations and normal CSF cell count. The median CSF CCL19 content of the control group IE was 66 pg/ml. Healthy dogs had a median CSF CCL19 concentration of 51.2 pg/ml (Table 1). The significant difference between IE and healthy dogs suggests that CCL19 might be involved in the pathway of IE. Since only a small number of neurologically normal dogs was available for evaluation, further studies with a larger cohort of dogs focusing on IE in comparison to healthy dogs are indicated.

### CCL19 CSF concentration in inflammatory diseases

**Figure 2:**
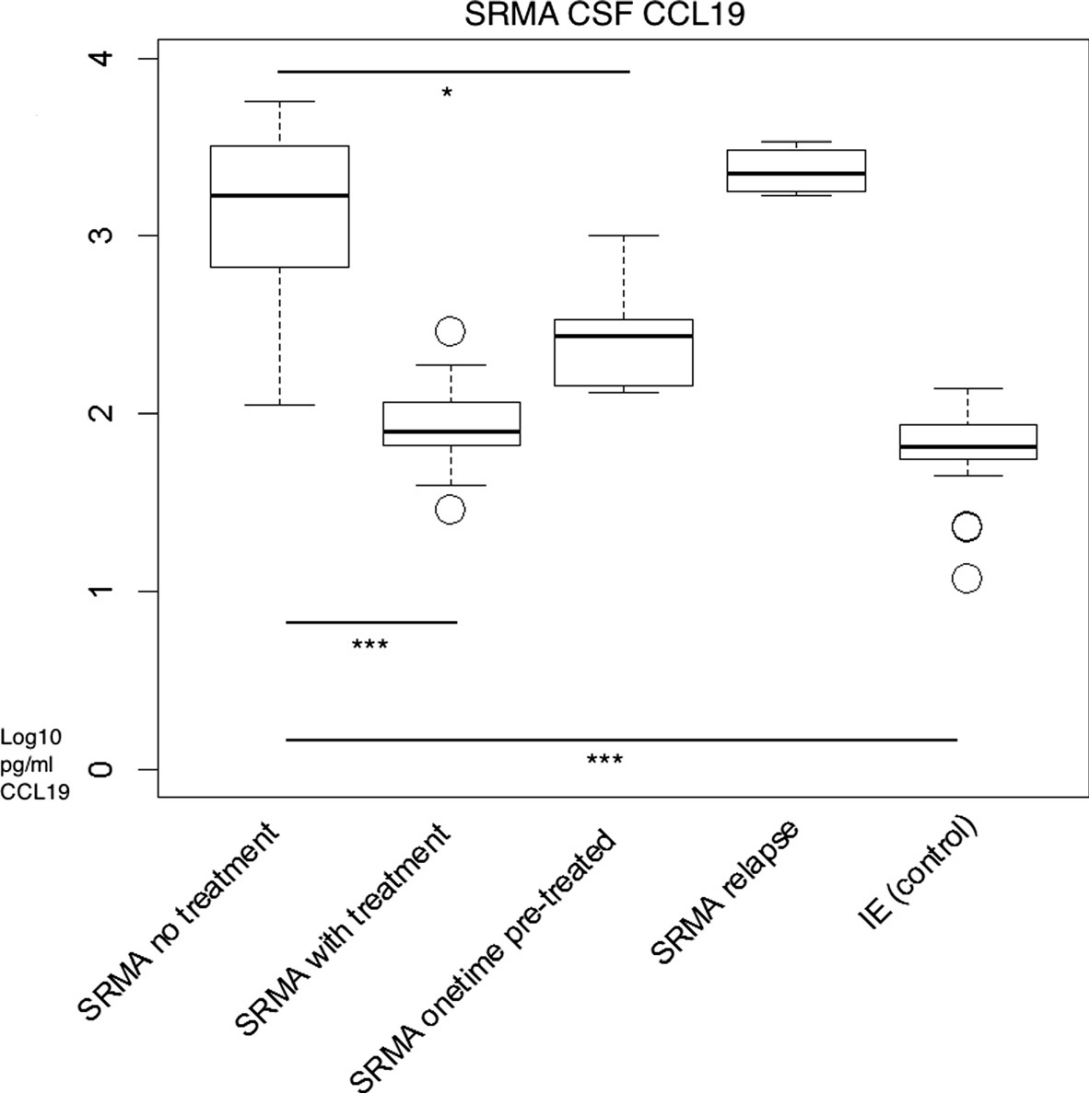
**CCL19 CSF concentrations of patients with SRMA compared to the control group.** y- axis: logarithm to base 10 of CCL19 (pg/ml); x- axis: groups. Boxes contain values from the 1st to the 3rd quartile, lines inside boxes indicate median values, endpoints of vertical lines display minimum and maximum values, o represents the outliers. Asterisks indicate statistically significant differences (*P < 0.05; **P < 0.01; ***P < 0.005). SRMA = steroid-responsive meningitis-arteritis; CSF = cerebrospinal fluid; IE = idiopathic epilepsy.

**Figure 3:**
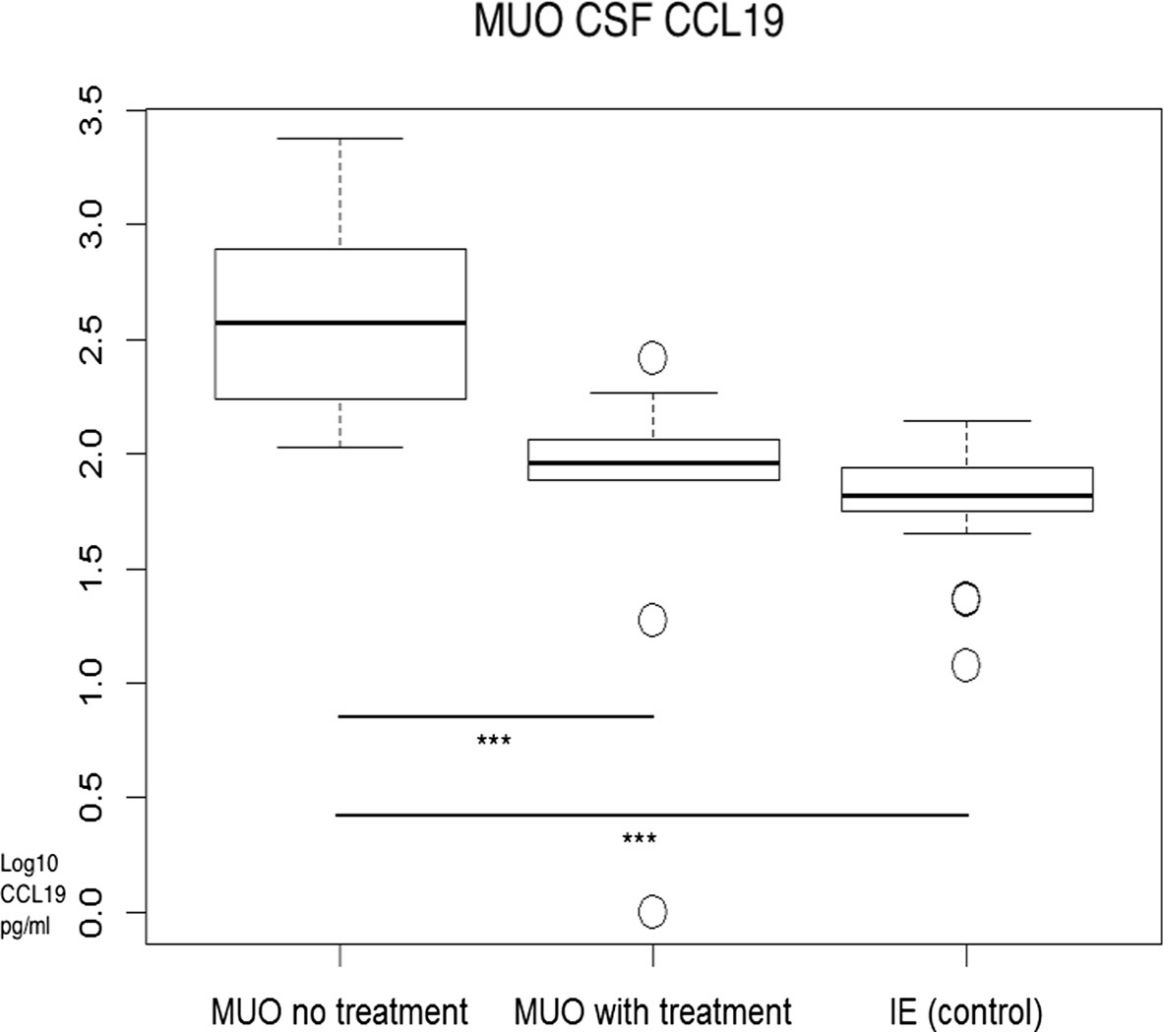
**CCL19 CSF concentrations of patients with MUO compared to the control group.** y- axis: logarithm to base 10 of CCL19 (pg/ml); x- axis: groups. Boxes contain values from the 1st to the 3rd quartile, lines inside boxes indicate median values, endpoints of vertical lines display minimum and maximum values, o represents the outliers. Asterisks indicate statistically significant differences (*P < 0.05; **P < 0.01; ***P < 0.005). MUO = meningoencephalomyelitis of unknown origin; CSF = cerebrospinal fluid; IE = idiopathic epilepsy.

Significantly elevated CCL19 concentrations were observed in CSF samples of untreated SRMA and MUO patients compared to the control group (IE) (p < 0.001; median SRMA: 1690 pg/ml; MUO: 373.5 pg/ml) as shown in Figure 2, Figure 3, and Table 1. Dogs that had undergone therapy had significantly lower CCL19 values compared to the patients without therapy (p < 0.001). Treated SRMA dogs (p = 0.1427) and treated MUO patients (p = 0.1368) did not differ significantly from the controls. SRMA patients, which received one injection of glucocorticosteroids before initial examination, had significantly lower CCL19 levels (p = 0.0259) compared to entirely untreated animals. The subgroup of patients having a relapse did not differ statistically (p = 0.4864) from untreated patients (Figure 2 and Figure 3).

### Further evaluation of inflammatory diseases

The CSF CCL19 concentrations of both untreated SRMA and untreated MUO patients were significantly correlated with the CSF cell count (SRMA r = 0.82, p < 0.001; MUO r = 0.59, p = 0.018).

SRMA and MUO patients under glucocorticosteroid treatment displayed no correlation between CSF cell count and CCL19 concentration as indicated by the results of Spearman and Kendall’s rank test (Spearman: SRMA r = 0.27, p = 0.19; MUO r = 0.53, p = 0.091). The CSF CCL19 and serum CCL19 concentrations did not correlate with each other indicating that CSF elevation is not due, at least entirely, to BBB disruption (Spearman: SRMA untreated r = 0.099, p = 0.64; MUO untreated r = 0.33, p = 0.22). Total protein levels within the CSF from untreated SRMA dogs had a weak correlation with CSF CCL19 concentrations using Spearman rank test (r = 0.39, p = 0.055); the Kendall rank test was performed due to the occurrence of outliers and revealed a significant correlation (tau = 0.33, p = 0.022). Dogs with MUO that did not receive therapy had no strong correlation between CCL19 CSF and CSF protein concentration (Spearman: r = 0.49, p = 0.057; Kendall: tau = 0.33, p = 0.079).

### CCL19 CSF concentration in intervertebral disc disease

**Figure 4:**
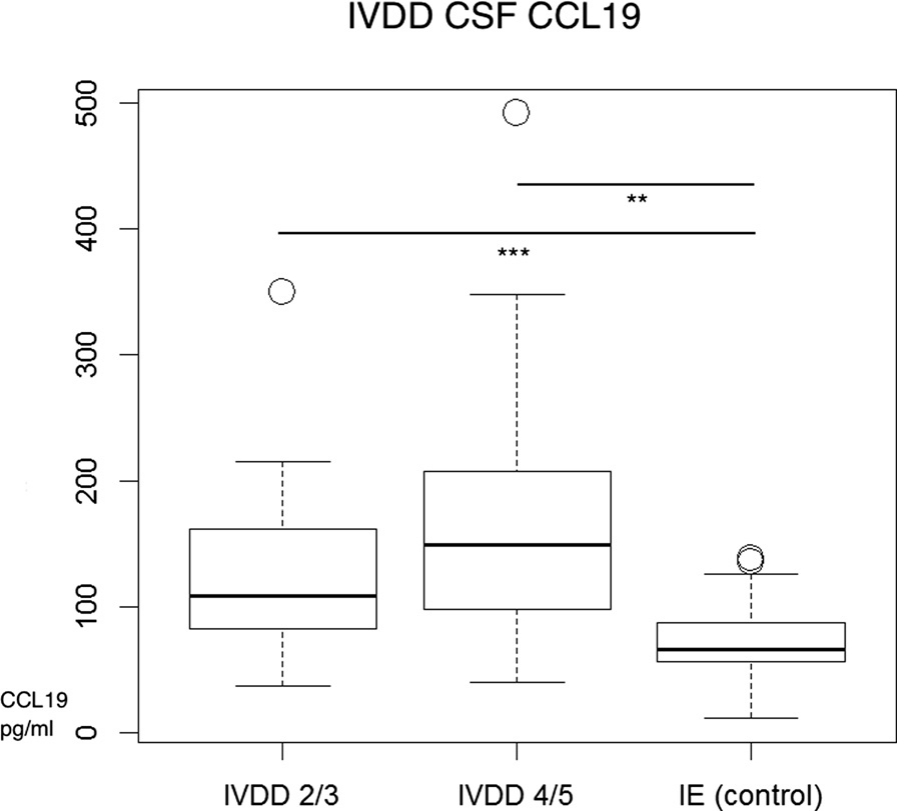
**CCL19 CSF concentrations of patients with IVDD compared to the control group.** Boxes contain values from the 1st to the 3rd quartile, lines inside boxes indicate median values, endpoints of vertical lines display minimum and maximum values, o represents the outliers. Asterisks indicate statistically significant differences (*P < 0.05; **P < 0.01; ***P < 0.005). IVDD = intervertebral disc disease; CSF = cerebrospinal fluid; IE = idiopathic epilepsy.

CSF CCL19 concentrations of IVDD are significantly elevated compared to the control group (p <0.05) as illustrated in the Box-and-Whisker Plots in Figure 4. The median concentration of the protein in the IVDD grade 2/3 group was 108.2 pg/ml while grades 4/5 had a higher median of 149.2 pg/ml, as compared to the controls with a median concentration of 66 pg/ml (Table 1). The concentrations between the two IVDD groups did not differ significantly (p = 0.26). In comparison to the median CCL19 concentrations from untreated inflammatory neurologic diseases (SRMA: 1690 pg/ml/MUO: 373.5 pg/ml) the elevations were mild (Figures 2, 3 and 4).

### Further evaluation of IVDD

No significant correlation of CSF CCL19 concentrations to CSF cell count was found among the IVDD group (Spearman: IVDD group 2/3 r = 0.05, p = 0.81; IVDD group 4/5 r 0.097, p = 0.78). CCL19 CSF concentration and CCL19 serum concentration were significantly correlated within IVDD group 2/3 (r = 0.44, p = 0.025) while IVDD group 4/5 had no significant correlation (r = 0.54, p = 0.094).

### Chemotactic activity of selected CSF samples for mononuclear cells

**Figure 5:**
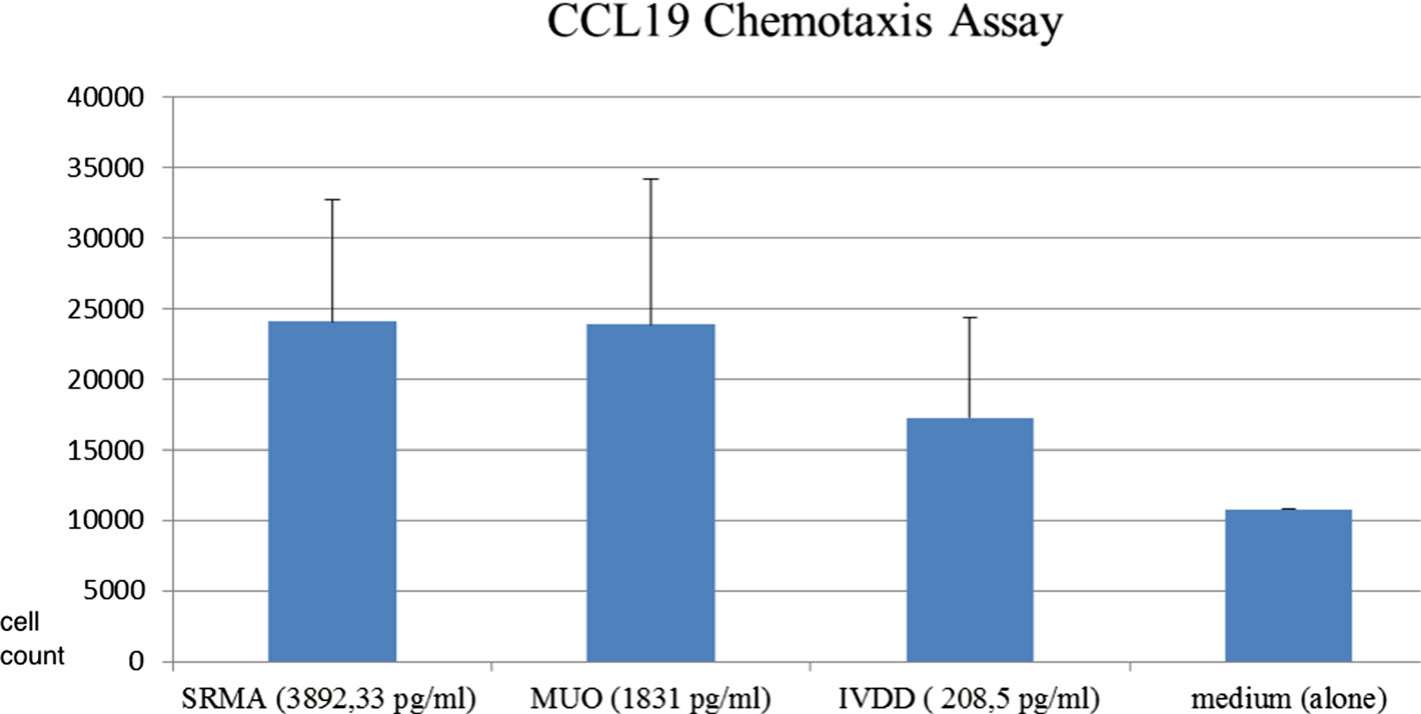
**Chemotaxis assay for mononuclear cells from CSF samples of SRMA, MUO, IVDD, medium.** Vertical lines indicate standard deviation. SRMA = steroid responsive meningitis-arteritis; MUO = meningoencephalomyelitis of unknown origin; IVDD = intervertebral disc disease; CSF = cerebrospinal fluid; pg = picogram; ml = milliliter.

CSF samples were used to induce mononuclear cell chemotactic activity. CSF of neuroinflammatory diseases, including SRMA and MUO, showed high cell migration as illustrated in Figure 5. The SRMA samples with a mean concentration of 3892.33 pg/ml CCL19 attracted 24083 cells and the MUO samples with a mean concentration of 1831 pg/ml CCL19 attracted 23876 cells after a two hour period, which was significantly more than the control group (medium 10785 cells). The CSF samples of dogs with IVDD with a much lower mean CCL19 concentration of 208.5 pg/ml compared to neuroinflammatory diseases also had a significant impact on migration and on average 17235 cells moved to the lower chamber after a 2 hour incubation time (Figure 5).

## Discussion

The results of this study support the hypothesis that the protein CCL19 is elevated in selected CNS diseases and might be involved in the pathogenesis of mononuclear cells migration into the subarachnoid space of dogs with inflammatory CNS diseases and non-inflammatory CNS diseases such as intervertebral disc disease (IVDD). CSF levels of the chemokine CCL19 were analyzed in dogs afflicted with SRMA, an inflammatory CNS disease, and compared to other neuroinflammatory diseases (MUO), traumatic IVDD, a non-inflammatory CNS disease, idiopathic epilepsy (IE) and normal unafflicted animals.

CCL19 was detectable in healthy dogs and in patients with idiopathic epilepsy, a non-inflammatory neurologic disease, which is in agreement with the results of other studies [11,16] in that CCL19 is constitutively expressed in the CNS and linked to the physiological immunosurveillance [11,16]. However, CCL19 was markedly increased in both neuro- inflammatory diseases SRMA and MUO (Figure 2 and 3). Thus, CCL19 might be involved in the pathogenesis of CNS inflammation and in the migration of inflammatory cells into the subarachnoid space. Krumbholz e*t al.*[11] and Pashenkov e*t al*. [16] observed a CSF CCL19 elevation in neuro- inflammatory diseases of human patients. Earlier observations have shown that CCL19 is also produced by neutrophils [34]. The higher concentration of the chemokine in SRMA is consistent with the fact that SRMA CSF has an increased neutrophilic inflammatory response. The elevated chemokine concentration may maintain the inflammatory response and later transfer to an autoimmune reaction attracting certain lymphocyte subsets. Additionally, a disrupted blood brain barrier is consistent with a strong inflammatory response and could support the explanation for much higher CCL19 data. However, CCL19 levels were higher in CSF samples compared to serum concentrations in neuroinflammatory diseases suggesting intrathecal production rather than passive diffusion through a disrupted blood brain barrier. Results of Pelletier *et al.*’s study revealed that a novel chemokine-dependent reciprocal cross-talk between neutrophils and Th17 cells exists [35]. Activated neutrophils induced chemotaxis of Th17 cells and in turn could attract more neutrophils [35]. Observations from Maiolini hint that the immunopathology of SRMA is maintained by a Th17 response [23]. Neutrophils can release a number of chemokines including CCL19, which can specifically induce chemotaxis of dendritic cells (DC) and trigger rapid integrin-dependent adhesion of CCR7-expressing lymphocytes [34]. The neutrophil’s capability of CCL19 production might be important in guiding these cells to the inflamed sites and contributing to the regulation of the immune response. It has been shown previously that CCR7 ligands like CCL19 have a function in stimulating DCs for IL-23 production and generation of pathogenic Th17 cells in EAE induction [36]. An uncontrolled interleukin (IL) IL-23–IL-17 pathway could maintain a chronic inflammatory response and lead to persistent immunopathology [24]. Investigating this relationship and uncovering other similar pathways could be vital to our understanding of the pathogenesis of chronic inflammatory diseases. The intrathecally produced CCL19 might not only play a role in inducing autoimmune diseases but also in maintaining chronic forms through recruitment of antigen presenting cells and lymphocytes, resulting in perpetual antigenic stimulation [37]. Guinti *et al.* also observed significant levels of CCL19 in patients affected with MS and patients affected with other neuroinflammatory diseases and suspect involvement in T-cell trafficking [38]. In our study, the strong correlation between intrathecal CCL19 and the CSF cell count suggest CCL19 may play an important role in recruiting immune cells to the CNS.

SRMA and MUO patients receiving glucocorticosteroids had significantly less CCL19 concentrations, showing that steroids have a strong impact in reducing the inflammatory pathway. Krumbholz *et al.*’s study did not reveal that MS patients receiving treatment differ from untreated patients [11]. Glucocorticosteroids are shown to reduce the invasion of neutrophils [39] and may alter the production of chemokines in dogs. Relapsing SRMA also displayed elevated chemokine concentrations. This finding suggests the potential utility of this protein for developing new treatment schemes in inflammatory diseases, and to indicate the success of treatment.

The elevation of CCL19 in IVDD CSF suggests a process of a secondary inflammatory response within the CNS [40]. Gray and white matter are affected by trauma, especially when white matter damage is observed [30,41] through apoptosis, necrosis, and inflammation. Increased inflammatory cytokines in patients with chronic spinal cord injury after trauma suggest an altered immunological activation. A study by Srugo *et al.* suggested CSF pleocytosis is positively associated with the severity of thoracolumbar spinal cord damage in dogs with IVDD. They found that the percentage of CSF macrophages can be used as a prognostic indicator for regaining ambulation in dogs that have lost deep pain sensation [31]. The current study addresses the question if CCL19 could also play an important role for the migration of cells and the inflammatory pathogenesis in IVDD. A modest correlation of CSF to serum CCL19 in IVDD group 2/3 suggest that CSF CCL19 is derived by leakage via extracellular pathways from the circulation and not solely from intrathecal production in this group of patients. However, no correlation was found in between CSF and serum CCL19 concentrations in IVDD group 4/5 and some samples showed much higher CSF values suggesting the likelyhood of intrathecal production. A set of chemokines is intrathecally produced in CNS diseases leading to an inflammatory response. The fact that CCL19 is significantly elevated but, did not correlate with CSF cell count in either IVDD group suggests that CCL19 is not solely responsible for the recruitment of cells but, may play a part in the pathogenesis of IVDD inflammation.

The performed chemotaxis assay from CSF samples attracted mononuclear cells. CSF of neuro-inflammatory diseases showed strong chemotactic activity in comparison to the controls. Even CSF samples of dogs with IVDD with a much lower mean CCL19 concentration showed a pronounced impact on cell migration, which suggests the presents of a functional chemotactic protein. Alt *et al*. showed that the expression of CCL19 occurs at the BBB in experimental autoimmune encephalomyelitis and concluded that this chemokine may contribute to T -cell attraction across the BBB [42]. CSF consists of various sets of chemokines responsible for cell migration. A chemotaxis study using a CCL19 antagonist may prove to be useful in defining CCL19’s role in mononuclear cell migration into the CNS of SRMA, MUO and IVDD dogs. A study from Pineau and Lacroix on rodent models revealed that cytokine production is time dependent [43]. CNS resident cells, including neurons, synthesized cytokines between 3 and 24 hours post SCI and some infiltrating leukocytes were responsible for the cytokine production 12 hours after the trauma. This would suggest that neural cells are responsible for the initial inflammatory response following SCI but, the recruitment of additional immune cells is responsible for the maintenance of inflammation [43]. Additional studies would be necessary to determine the time at which CCL19 becomes a major inflammatory mediator. Kwon *et al*. observed in his study that interleukin (IL)-6, IL-8, tau, S100b and glial fibrillary acidic protein were elevated depending on severity of the initial injury. Evaluating this group of inflammatory cytokines, a biochemical model was developed enabling veterinary scientists to predict the ASIA (American Spinal Injury Association) grade in 89% of their subjects. It was found that the pattern of protein expression within the CSF over the first few days following injury were strong indicators of neurologic outcome and thus, are now a focus of therapeutic research for SCI [44]. Using CCL19 CSF concentration as a prognostic marker could be also helpful in the research of SCI in dogs, which should be followed up in further studies.

## Conclusions

In conclusion, CCL19 CSF concentrations were markedly elevated in patients with neuroinflammatory disease and may play an important role in their pathogenesis with recruitment of inflammatory cells into the subarachnoid space of dogs. This study also showed an elevated CSF CCL19 concentration in dogs with IVDD, indicating that the chemokine might also be associated with the secondary wave of SCI, following an acute cord insult. A marked chemotactic activity for mononuclear cells was observed in selected CSF samples of the neuroinflammatory and non-neuroinflammatory disease. Further functional studies could confirm that CCL19 has a major role of mononuclear cell migration into the subarachnoid space of dogs affected with SRMA, MUO, IVDD and may be a precious target for developing new treatment schemes.

## Abbreviations

ASIA: American spinal injury association; BBB: Blood brain barrier; BSA: Bovines serum albumin; CCL19: C-C motif ligand 19; CCR7: C-C chemokine receptor; CNS: Central nervous system; CSF: Cerebrospinal fluid; DC: Dendritic cells; EAE: Chronic experimental autoimmune encephalomyelitis; FLAIR: “Fluid attenuated inversion recovery”; GME: Granulomatous meningoencephalomyelitis; ELISA: Enzyme linked immunosorbent assay; HBSS: Hank’s balanced salt solution; IE: Idiopathic epilepsy; Ig: Immunoglobulin; IL: Interleukin; IVDD: Intervertebral disc disease; log: Logarithmus; MIP-3β: Macrophage inflammatory protein-3beta; ml: Milliliter; MRI: Magnetic resonance imaging; MUO: Meningoencephalomyelitis of unknown origin; μl: microL, microliter; mg: Milligram; ml: Milliliter; NE: Necrotizing encephalitides; No: Number; PBS: Phosphate buffered saline; PMC: Peripheral mononuclear cells; pg: Picogram; RPMI: Rosewell park memorial institute; SCI: Spinal cord injuries; SRMA: Steroid responsive meningitis-arteritis; TMB: 3,3′,5,5′-Tetramethylbenzidine; VESC: Veterinary Emergency and Specialty Center.

## Competing interests

The authors declare that they have no competing interests.

## Authors’ contributions

JB designed and performed the ELISA assay experiments as well as the chemotaxis assay experiments and drafted the manuscript. BD participated in the analysis of the study and helped to draft the manuscript. SJS provided the samples and participated in the coordination of the study. LB performed the statistical analysis. RC helped with the execution of laboratory methods. AT conceived of the study, participated in the design and coordination of the study and helped to draft the manuscript. All authors read and approved the final manuscript.
